# A ten-year China-US laboratory collaboration: improving response to influenza threats in China and the world, 2004–2014

**DOI:** 10.1186/s12889-019-6776-3

**Published:** 2019-05-10

**Authors:** Yuelong Shu, Ying Song, Dayan Wang, Carolyn M. Greene, Ann Moen, C. K. Lee, Yongkun Chen, Xiyan Xu, Jeffrey McFarland, Li Xin, Joseph Bresee, Suizan Zhou, Tao Chen, Ran Zhang, Nancy Cox

**Affiliations:** 1 Chinese National Influenza Center, National Institute for Viral Disease Control and Prevention, Collaboration Innovation Center for Diagnosis and Treatment of Infectious Diseases, Chinese Center for Disease Control and Prevention, Key Laboratory for Medical Virology, National Health and Family Planning Commission, Beijing, 102206 People’s Republic of China; 2 Influenza Division, U.S. Centers for Disease Control and Prevention, WHO Collaborating Center for Surveillance, Epidemiology and Control of Influenza, Atlanta, GA 30333 USA; 3 On behalf of Emerging Disease Surveillance and Response (ESR), World Health Organization Western Pacific Region, Manila, Philippines

**Keywords:** Influenza, Laboratory, China, International cooperation, Surveillance

## Abstract

The emergence of severe acute respiratory syndrome (SARS) underscored the importance of influenza detection and response in China. From 2004, the Chinese National Influenza Center (CNIC) and the United States Centers for Disease Control and Prevention (USCDC) initiated Cooperative Agreements to build capacity in influenza surveillance in China.

From 2004 to 2014, CNIC and USCDC collaborated on the following activities: 1) developing human technical expertise in virology and epidemiology in China; 2) developing a comprehensive influenza surveillance system by enhancing influenza-like illness (ILI) reporting and virological characterization; 3) strengthening analysis, utilization and dissemination of surveillance data; and 4) improving early response to influenza viruses with pandemic potential.

Since 2004, CNIC expanded its national influenza surveillance and response system which, as of 2014, included 408 laboratories and 554 sentinel hospitals. With support from USCDC, more than 2500 public health staff from China received virology and epidemiology training, enabling > 98% network laboratories to establish virus isolation and/or nucleic acid detection techniques. CNIC established viral drug resistance surveillance and platforms for gene sequencing, reverse genetics, serologic detection, and vaccine strains development. CNIC also built a bioinformatics platform to strengthen data analysis and utilization, publishing weekly on-line influenza surveillance reports in English and Chinese. The surveillance system collects 200,000–400,000 specimens and tests more than 20,000 influenza viruses annually, which provides valuable information for World Health Organization (WHO) influenza vaccine strain recommendations. In 2010, CNIC became the sixth WHO Collaborating Centre for Influenza. CNIC has strengthened virus and data sharing, and has provided training and reagents for other countries to improve global capacity for influenza control and prevention.

The collaboration’s successes were built upon shared mission and values, emphasis on long-term capacity development and sustainability, and leadership commitment.

## Background

The Chinese National Influenza Center of the Chinese Center for Disease Prevention and Control (China CDC) and the Influenza Division of the United States Centers for Disease Control and Prevention first discussed influenza virological surveillance in China in 1978. In 1989, CNIC and USCDC signed agreements that enabled USCDC to provide technical and financial support for influenza surveillance in China and laid the foundation for future collaborations between the two agencies. Initially, USCDC provided funds directly to CNIC for laboratory training and specimen sharing; later, funding was provided through WHO headquarters, and subsequently through the WHO Western Pacific Regional Office. Between 1989 and 2004, CNIC and USCDC staff traveled through WHO. Between 1989 and 2004, CNIC and USCDC staff traveled to each other’s agencies for training and collaborative studies.

In 2000, China enhanced earlier influenza surveillance efforts by establishing an influenza-like illness (ILI) and virological surveillance system to report ILI cases and isolate viruses for seasonal influenza vaccine strain recommendations. The surveillance system, including 8 network laboratories and 31 sentinel hospitals, did not capture the diversity of influenza activity and viruses circulating throughout the country. In addition, China recognized that the poor quality of the data with respect to completeness, timeliness and accuracy, limited the system’s capacity to contribute to public health practice. The emergence of SARS in 2003 [1] and avian influenza A (H5N1) virus in 2005 in mainland China [2] underscored China’s role as a potential source for emerging novel influenza viruses, due to its large human population, extensive and rapidly expanding poultry and swine production, cultural practices that increase exposures at the human-animal interface, and consumer preference for live poultry [3]. Recognizing the importance of high-quality influenza surveillance in China, from 2004, China CDC and USCDC established influenza and global disease detection (GDD) Cooperative Agreements to improve the ILI and virological surveillance system in mainland China, and to expand CNIC’s role from contributing to seasonal influenza vaccine strain recommendations to conducting early detection and response to novel influenza viruses with pandemic potential.

In this report, we review the China-US laboratory collaboration on influenza from 2004 to 2014, to share best practices and lessons learned, and to assess how this collaboration builds capacity in the prevention and control of influenza in China and globally.

### The China-US influenza collaboration from 2004 to 2014

Building upon existing technical exchanges and training programs, CNIC and the Influenza Division of the USCDC signed a bilateral Cooperative Agreement in 2004 to develop and build the capacity of the influenza surveillance laboratory networks in China. In 2007, China CDC and USCDC signed an additional Cooperative Agreement on emerging and remerging infectious diseases which was funded by the USCDC GDD program that allowed for enhanced collaborations with CNIC in the field of influenza. The objectives for these collaborations were: 1) to improve and expand the influenza surveillance system in China; and 2) to build capacity for early detection and response to seasonal influenza, avian influenza and other influenza viruses with pandemic potential. In September 2007, the USCDC deployed the first influenza assignee to China and established a USCDC program team in China, comprised of both a USCDC staff member from the United States and one to three locally-employed staff, to ensure effective communication and cooperation between CNIC and USCDC. Members of the USCDC team in country and colleagues from the Influenza Division in Atlanta worked closely with CNIC on a routine basis and contributed to numerous accomplishments including: 1) developing human technical expertise in virology and epidemiology in China; 2) improving the quality and function of the influenza surveillance system in China; 3) strengthening the analysis, utilization and dissemination of surveillance data; and 4) improving early response to avian influenza and other influenza viruses with pandemic potential. The following collaborative activities were identified to meet these priorities: 1) training and enhancing human workforce development in CNIC and network laboratories across the country; 2) strengthening quality improvement activities including a) improving the quality of influenza virological surveillance through nucleic acid detection, virus isolation in embryonated eggs, genetic and antigenic characterization, and receptor-binding specificity characterization; b) strengthening quality assurance of network laboratories by conducting annual laboratory quality assessments; c) initiating the ISO15189 accreditation program; and d) introducing new technology including Nucleic Acid Sequence Based Amplification (NASBA), multi-plex PCR, and deep sequencing in CNIC; 3) adding new functions to the influenza surveillance system including: drug resistance surveillance; monitoring serologic status among occupationally exposed groups; conducting environmental sampling surveillance; and using multi-pathogen detection platforms for the surveillance of other respiratory viruses; 4) establishing an influenza information system to integrate epidemiology and laboratory data and to promote timely data analysis and sharing; and 5) promoting international collaborations in the form of information exchanges and virus-sharing during the 2009 influenza pandemic and the influenza A(H7N9) outbreaks.

1. Developing human technical expertise in virology and epidemiology in China

From 2004 to 2014, 15 CNIC staff received training in CDC Atlanta, from one month to 1.5 years in length, on topics including influenza surveillance and laboratory management, reverse genetic techniques, serological techniques, pathology, antigenicity characterization, and drug resistance surveillance. CNIC provided lecture-based training to 2320 staff from network laboratories and sentinel hospitals, and hands-on training to 450 lab specialists on cell culture, virus isolation, and serology testing and gene sequence analysis with USCDC support.

2. Improving the quality and function of the influenza surveillance system in China

**Fig. 1:**
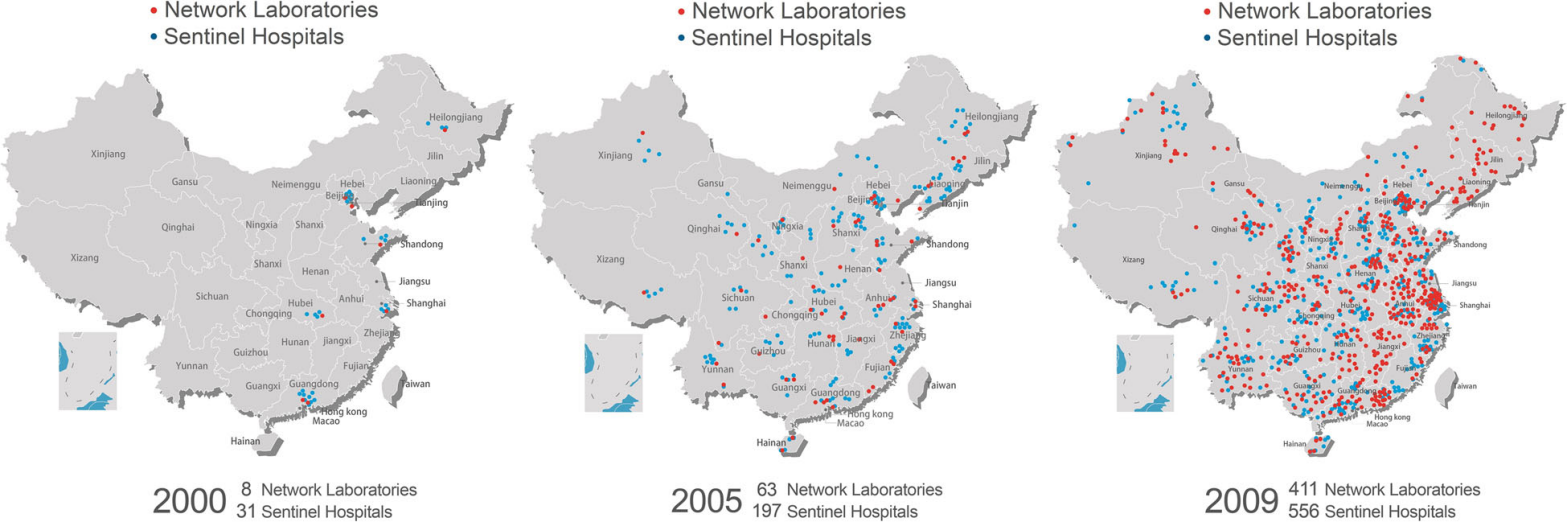
Influenza network laboratories and sentinel hospitals in Mainland China, 2000–2009. Figure created by the Chinese National Influenza Centre authors

**Fig. 2:**
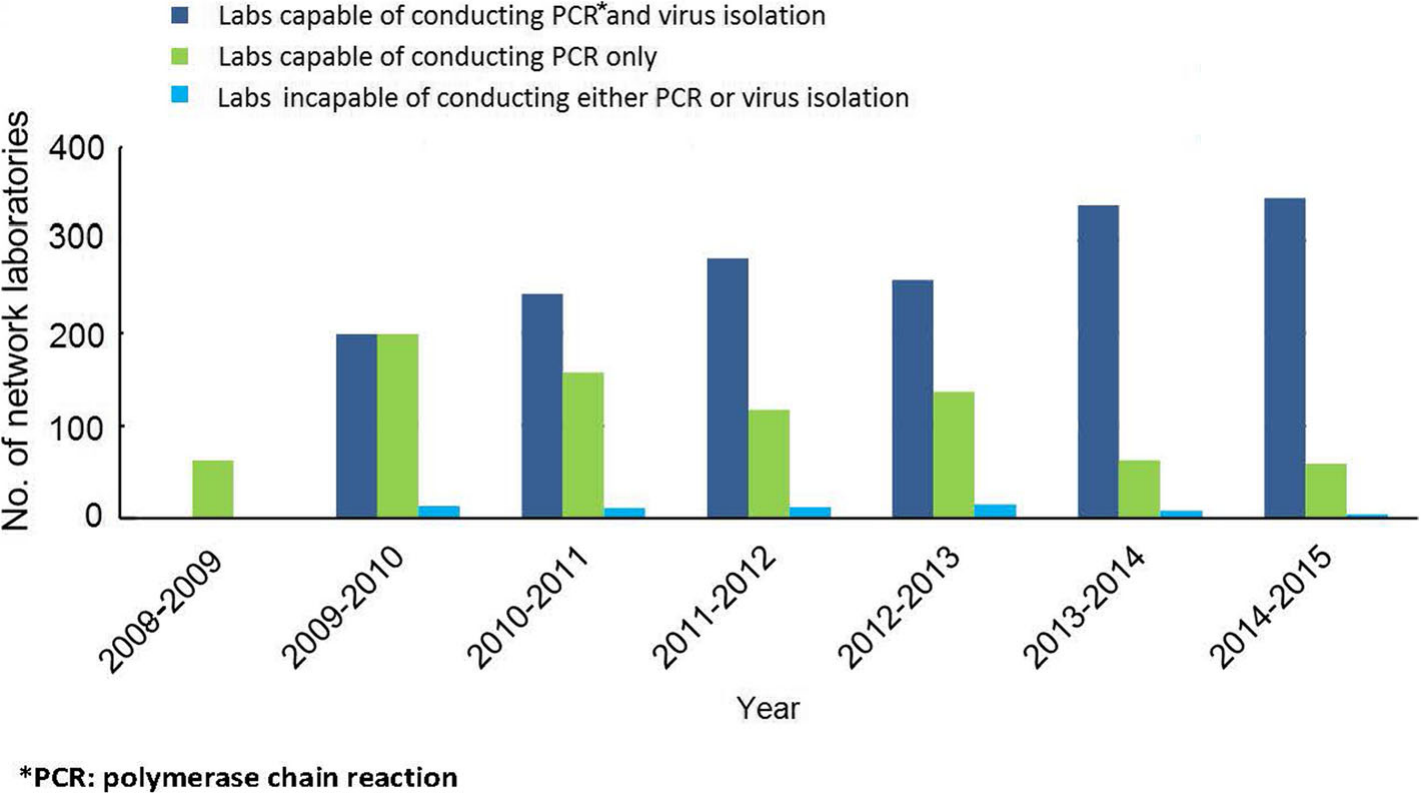
Number of network laboratories capable of conducting virus isolation, China, 2008–2015

During this timeframe, China also expanded its ILI and virological surveillance network. In 2005, the network increased from 8 to 63 network laboratories and 31 to 197 sentinel hospitals, primarily supported with funds from the Chinese government, with supplemental project-based financial support from USCDC and WHO. During the 2009 influenza A(H1N1) pandemic period, the Chinese government expanded the network further to include 411 laboratories and 556 sentinel hospitals (Fig. 1). The China-US collaboration, complementing the national program, aimed to strengthen capacity by providing training, strengthening laboratory quality assessment and accreditation, and establishing new laboratory testing technologies. From 2012 to 2014, 21 provincial influenza labs were certificated by CNIC as Provincial Influenza Reference Centers. The number of network laboratories capable of performing nucleic acid detection increased from 63 in 2005 to 403 in 2014, and more than 80% of all 408 network laboratories were capable of performing virus isolation (Fig. 2). Similarly, the number of network laboratories with capacity to conduct virus isolation in eggs to support the selection of vaccine viruses increased from 66 in 2009 to 140 in 2014.

**Fig. 3:**
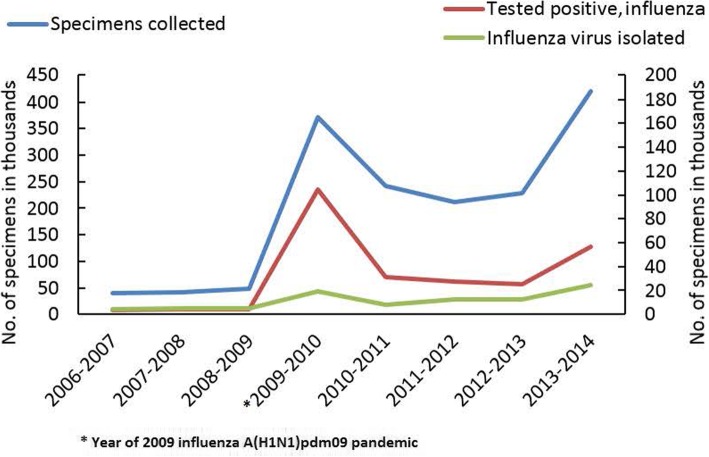
Number of respiratory specimens collected, tested positive for influenza, and influenza viruses isolated from influenza surveillance network, China, 2006–2014

From 2006 to 2014, the network laboratories substantially increased the number of specimens processed annually from 38,039 to 455,180, the number of specimens tested for influenza viruses by real-time RT-PCR from 0 to 397,150, and the number of influenza viruses isolated from specimens collected within the influenza surveillance system from 3565 to 28,685 (Fig. 3).

Over the ten-year period, CNIC conducted full genome sequencing on 4039 influenza viruses, the number of viruses sequenced per year increased from 225 in 2004 to 334 in 2014. With technical support from USCDC, CNIC established surveillance for anti-viral drug resistance in 2004. A total of 11,192 viruses of different types/subtypes were analyzed during the ten years, and the number of strains tested for drug susceptibility increased over 100-fold, from 31 in 2004 to 3929 in 2014 [4–11].

From 2008 to 2014, CNIC conducted annual quality assessments to monitor the quality of specimen collection, storage, transportation and testing of the network laboratories. The proportion of network laboratories capable of achieving 100% correct test results for RT-PCR and real-time RT-PCR increased from 87% in 2008 to 96% in 2014 (Table 1). In addition, CNIC participates in WHO’s annual external quality assurance program (EQAP), and has received 100% accurate results since 2007.

**Table 1:** Nucleic acid assessment of municipal/district/county-level network CDC influenza laboratories, China, 2008–2014^1^

	2008	2009	2010	2011	2012	2013	2014^2^
Number of network laboratory at municipal (district or county) level participating in the assessment	31	367	372	370	370	371	243
Number of CDCs with completely correct assessment results	27	293	299	359	342	356	234
% of participating CDCs with completely correct results	87	80	80	97	92	96	96

^1^Assessment conducted by the Chinese National Influenza Center (CNIC). CNIC evaluates the testing results of participating network laboratories

^2^Starting in 2014, the national assessment only included one network laboratory from the provinces designated as provincial reference centers; the remaining laboratories within these provinces participated in the provincial assessments (not shown here)

A year after WHO’s assessment of CNIC in 2007, the Chinese Ministry of Health submitted an application for CNIC to become a WHO Collaborating Centre (WHO CC) for Reference and Research on Influenza. CNIC implemented performance improvement measures based on WHO requirements, and launched a one-year assessment in November 2008. In October 2010, CNIC was officially designated as the world’s sixth WHO CC for Influenza, joining laboratories in Australia, Japan, the United Kingdom and the United States.

As the ILI surveillance system expanded in size and capacity, its function also expanded to the early detection of emerging novel influenza viruses. Built on the existing influenza surveillance network, CNIC developed an influenza identification platform which can detect all types/subtypes of influenza viruses including zoonotic infections. This system allowed CNIC to confirm China’s first human infections from A (H7N9), A (H10N8) and A (H5N6) viruses just 1–3 days after receiving specimens [12–14].

3. Strengthening the analysis and dissemination of surveillance data

CNIC developed an information system with three online components: the Influenza Surveillance Information System, the Infectious Disease Surveillance Platform and the Influenza Prediction and Early Warning Platform. Local CDC users across China can access this system to: 1) report cases of ILI, pneumonia of unknown etiology, and severe acute respiratory infection (SARI); and 2) upload laboratory testing results. The Infectious Disease Surveillance Platform also collects test results from environmental surveillance and serologic studies of groups with occupational exposure to poultry. With support from the collaboration with USCDC strengthened data analysis and, since 2009, has generated online Weekly Influenza Reports to share when, where and which influenza viruses are circulating in China. The weekly reports, in both Chinese and English, are emailed to key stakeholders and are also made available on the CNIC website [15].

Since 2005, CNIC also has reported influenza surveillance data to WHO FluNet [16]. During the influenza A(H7N9) virus outbreak, CNIC detected and reported the first case of human infection with A(H7N9) virus to WHO on March 31, 2013 within one week of receiving the specimen. The first paper describing this case was published in *The New England Journal of Medicine* 11 days after case confirmation [14]. Subsequently, CNIC has published more than 15 additional peer-reviewed papers on A(H7N9) virus infections in humans to describe the virological characteristics of A(H7N9) virus [17–30] for the international public health audience, and to inform A(H7N9) outbreak response efforts.

4. Improving early response to novel influenza viruses with pandemic potential

During the early stages of the 2009 H1N1 pandemic, USCDC and China CDC established routine conference calls between directors and experts of the two centers. USCDC shared the genetic sequence of the pandemic virus with CNIC, allowing CNIC to rapidly develop nucleic acid detection kits which were transported to national influenza network laboratories and other laboratories across the country. The availability of testing reagents ensured accurate estimates of the magnitude of the pandemic in China and allowed the Chinese government to coordinate an appropriate response. In addition, the USCDC country team in Beijing worked closely with China CDC experts on pandemic risk assessment and response.

In 2013, CNIC shared novel avian influenza A (H7N9) viruses from human infections with WHO Collaborating Centers and other qualified laboratories worldwide. CNIC also worked with WHO, USCDC and other international laboratories to modify serological protocols for influenza A (H7N9) virus detection [31]. International collaboration for the first human outbreak of avian influenza A (H7N9) virus was exemplified by a joint mission of Chinese and international influenza experts to Beijing and Shanghai from 18 to 24 April 2013. Team members included representatives from China’s National Health and Family Planning Commission (NHFPC), China CDC, international influenza experts from Australia, Europe, Hong Kong (China), the US, and WHO. The mission report, covering recommendations for ongoing surveillance and investigations, information sharing and collaboration, and preparedness and response, was released and shared through WHO and NHFPC websites. In addition, two USCDC epidemiologists from traveled to China, to work with China CDC experts to analyze surveillance data, design case control and serologic study protocols, and discuss response measures. At the same time, two CNIC senior staff spent 3 months at US CDC’s Influenza Division to receive training on deep sequencing of influenza A(H7N9) viruses.

5. Promoting international collaboration and cooperation

As a new WHO Collaborating Centre for Influenza, CNIC not only increased influenza control and prevention capacity throughout China, but also throughout the region. In recent years, CNIC, with Chinese government support, has provided hands-on laboratory training to neighboring countries, such as from the Association of Southeast Asian Nations (ASEAN). More specifically: in 2007, CNIC provided avian influenza laboratory diagnostic training to 18 trainees from 9 ASEAN countries; during the 2009 pandemic, CNIC provided free test kits to 13 countries and training on biosafety and laboratory diagnosis of A(H1N1pdm09) virus for 16 participants from 8 ASEAN countries; and in 2013, CNIC conducted training on serological detection of avian influenza A(H7N9) virus for 7 participants from Indonesia, Laos, Malaysia, Mongolia, Philippines, Thailand and Vietnam. Further, CNIC shared serological and nucleic acid testing protocols with WHO and provided diagnostic kits for pandemic H1N1 2009 to Brunei, Cambodia, Cuba, Indonesia, Laos, Malaysia, Mongolia, Papua New Guinea, Philippines, Singapore, Thailand and Vietnam, and seasonal influenza surveillance-related reagents and consumables to DPR Korea.

### What we learned from the collaboration

In the last decade, the CNIC-USCDC collaboration has built capacity in seasonal and novel influenza prevention and control in China and beyond. The workforce of the influenza surveillance network, both within CNIC and within network laboratories and sentinel hospitals throughout China, is now well developed. The influenza surveillance system expanded in size, capacity, and function, and improved the quality of its testing and use of data. No longer focused on seasonal influenza alone, the ILI surveillance network detected the first human infections with influenza A (H7N9) virus, A (H10N8) virus, A (H5N6) virus and other novel avian influenza viruses. The collaboration promoted timely data analysis and sharing, including the development of an on-line influenza weekly report for early case reporting and information sharing. In addition, the collaboration enabled CNIC to provide technical assistance in the field of influenza laboratory diagnostics to numerous countries in the region. One challenge faced by the CNIC-USCDC collaboration during the past decade was the limited effective coordination with animal health sectors in the field of avian influenza in China. In the future, the two agencies plan to strengthen avian influenza collaborations within a one-health approach that promotes a multi-sectoral response to emerging influenza threats. A major contributor to the success of this collaboration was that it complemented the existing national influenza program, in addition to the Chinese government’s commitment to developing an extensive and robust influenza surveillance system. In 2009, the Chinese government invested the equivalent of 46 million US dollars to expand the network. All collaborative efforts were designed to support the successful expansion of the network. For example, to ensure the quality of the new system, the CNIC-USCDC collaboration provided training for laboratory specialists within the new network laboratories and sentinel hospitals and improved quality assessment of the system. The collaboration also supported the establishment of new components of the surveillance system, such as antiviral resistance surveillance and environmental surveillance. At the outset, CNIC and USCDC agreed that these new components would soon be fully incorporated and maintained by the national program. In this way, the collaboration maximized existing government investments in influenza surveillance. Further, the collaboration demonstrated the importance of influenza surveillance during response efforts to the 2009 pandemic and the 2013 A(H7N9) outbreak, ensuring continued government commitment to the influenza surveillance system in the future.

The CNIC-USCDC collaboration was strengthened by the two agencies’ shared mission and values. Long before the launch of the official cooperative agreement in 2004, both agencies recognized the importance of improving influenza surveillance in China; the recent A(H5N1) and SARS outbreaks highlighted China as a potential source for emerging novel respiratory viruses with pandemic potential. Further, USCDC’s strategic priorities at the time included increasing global surveillance and response capacity to strengthen global health security and China, the most populous country in the world, played a critical role. In this way, the collaboration between CNIC and USCDC was founded on a joint mission and mutual, well-defined priorities.

Another factor contributing to the success of the collaboration was its commitment to capacity building. CNIC plays a key role managing and developing the influenza surveillance network in China, and therefore, CNIC staff members who received training at USCDC and other international organizations played a vital role during the responses to both the 2009 A(H1N1) pandemic and the 2013–2014 A(H7N9) outbreaks in terms of early virus identification, deep gene sequencing, vaccine development, and serving as trainers in both domestic and international settings.

Finally, this CNIC-USCDC collaboration would not have thrived without the shared vision and commitment of the leaders within the two agencies. Fortunately, the formal Cooperative Agreement was built upon the foundation of a long-term collaboration between CNIC and USCDC. In the decades prior to 2004, influenza experts from both agencies had developed strong professional respect for one another, as they shared a profound interest and commitment to preventing and controlling influenza illness around the globe. The leadership’s respect and trust for one another likely facilitated the rapid joint responses to emergencies, which included data and virus sharing, technical exchanges, and joint manuscripts in the peer-reviewed literature.

## Conclusions

In the past decade, China rapidly expanded its capacity to detect and respond to seasonal influenza and novel influenza viruses with pandemic potential. The 2004–2014 China-US collaboration in the field of influenza demonstrates how two public health agencies worked together to expand a disease surveillance system, and ultimately improved the prevention and control of both seasonal and novel influenza viruses in China and the world.

## Abbreviations

ASEAN: Association of Southeast Asian Nations
China CDC: Chinese Center for Disease Prevention and Control
CNIC: Chinese National Influenza Center
EQAP: External Quality Assurance Program
GDD: Global Disease Detection
ILI: Influenza-like Illness
NHFPC: National Health and Family Planning Commission
SARS: Severe Acute Respiratory Syndrome
USCDC: United States Centers for Disease Control and Prevention
WHO CC: WHO Collaborating Centre
WHO: World Health Organization
